# A methodological framework for the evaluation of syndromic surveillance systems: a case study of England

**DOI:** 10.1186/s12889-018-5422-9

**Published:** 2018-04-24

**Authors:** Felipe J. Colón-González, Iain R. Lake, Roger A. Morbey, Alex J. Elliot, Richard Pebody, Gillian E. Smith

**Affiliations:** 1 School of Environmental Sciences, University of East Anglia, Norwich, NR4 7TJ UK; 2 Real-time Syndromic Surveillance Team, National Infection Service, Public Health England, Birmingham, B3 2PW UK; 3 Respiratory Diseases Department, National Infection Service, Public Health England, London, NW9 5EQ UK; 4 NIHR Health Protection Research Unit for Emergency Preparedness and Response, London, UK

**Keywords:** Syndromic surveillance, Scenarios, Simulation, Influenza, Cryptosporidiosis

## Abstract

**Background:**

Syndromic surveillance complements traditional public health surveillance by collecting and analysing health indicators in near real time. The rationale of syndromic surveillance is that it may detect health threats faster than traditional surveillance systems permitting more timely, and hence potentially more effective public health action. The effectiveness of syndromic surveillance largely relies on the methods used to detect aberrations. Very few studies have evaluated the performance of syndromic surveillance systems and consequently little is known about the types of events that such systems can and cannot detect.

**Methods:**

We introduce a framework for the evaluation of syndromic surveillance systems that can be used in any setting based upon the use of simulated scenarios. For a range of scenarios this allows the time and probability of detection to be determined and uncertainty is fully incorporated. In addition, we demonstrate how such a framework can model the benefits of increases in the number of centres reporting syndromic data and also determine the minimum size of outbreaks that can or cannot be detected. Here, we demonstrate its utility using simulations of national influenza outbreaks and localised outbreaks of cryptosporidiosis.

**Results:**

Influenza outbreaks are consistently detected with larger outbreaks being detected in a more timely manner. Small cryptosporidiosis outbreaks (<1000 symptomatic individuals) are unlikely to be detected. We also demonstrate the advantages of having multiple syndromic data streams (e.g. emergency attendance data, telephone helpline data, general practice consultation data) as different streams are able to detect different outbreak types with different efficacy (e.g. emergency attendance data are useful for the detection of pandemic influenza but not for outbreaks of cryptosporidiosis). We also highlight that for any one disease, the utility of data streams may vary geographically, and that the detection ability of syndromic surveillance varies seasonally (e.g. an influenza outbreak starting in July is detected sooner than one starting later in the year). We argue that our framework constitutes a useful tool for public health emergency preparedness in multiple settings.

**Conclusions:**

The proposed framework allows the exhaustive evaluation of any syndromic surveillance system and constitutes a useful tool for emergency preparedness and response.

**Electronic supplementary material:**

The online version of this article (10.1186/s12889-018-5422-9) contains supplementary material, which is available to authorized users.

## Background

Syndromic surveillance collects, analyses, interprets and disseminates health-related data to provide early warnings about public health threats in near-real-time [1]. The original focus of syndromic surveillance was on both potential covert release of a disease causing agent, as well as monitoring of the more established risks of emerging infectious disease outbreaks. A key rationale of syndromic surveillance is that it may detect health threats faster than traditional surveillance systems, such as laboratory reports, which may permit more timely, and hence potentially more effective public health action to reduce morbidity and mortality [2]. Syndromic surveillance may help provide situational awareness, determining the trends, size, spread, and tempo of outbreaks, or provide reassurance that significantly large outbreaks are not occurring [3].

Syndromic surveillance systems are often used within national surveillance programmes, to detect a wide variety of events. In this study, we define detection as the identification of anomalous patterns (aberrations) in one or more syndromic indicators (e.g. increases in shortness of breath) within one or more syndromic data streams (e.g. emergency department attendances or telehealth calls) [4]. This is common to most papers on syndromic surveillance detection. Following detection of such an anomalous statistical pattern, in most cases this would be evaluated further bringing in additional information such as laboratory test data and medical intelligence methods to evaluate whether the detection is of public health relevance. The process through which this is achieved is described elsewhere for England [5]. In this paper we focus purely upon the detection of an anomalous statistical pattern in the syndromic indicators.

The main challenge in developing the analytical and statistical methods that underpin syndromic surveillance systems is detecting signals of anomalous activity in the presence of substantial background noise [3]. This is usually achieved using statistical methods, which use historical or recent data to determine statistical limits within which usual activity is expected to fall. A data value outside these bounds (exceedance) is a marker of potential unusual activity that might require further investigation. However, many of these exceedances may be random events of no public health consequence. Therefore, any statistical exceedances are usually rigorously examined before any public health action is taken [5]. For context, in England only around 0.8% of all statistical exceedances result in public health action.

To fully evaluate the public health role of syndromic surveillance, it is critical to assess the types of events that can be detected, how long such systems take to detect the event, and (of equal importance) those events that cannot be detected. For example, an outbreak of cryptosporidiosis may only be detected if a minimum number of cases access health care services, and this may vary between different geographical locations.

Research evaluating the performance of syndromic surveillance systems is scarce and has typically used a single disease type [6, 7], and one or two syndromic data sources [8–11]. This knowledge gap is important to address because different diseases and data streams are likely to exhibit different behaviours (see for example [12, 13]). In some studies, single day outbreak data were used to evaluate the detection performance of aberration detection methods [14, 15] which conflicts with the real behaviour of epidemics [8]. Some studies have simulated syndromic data [10, 16] which raises questions as to how well such data represent real trends in syndromic time series. One major limitation of previous studies is that they rarely incorporate uncertainty into their analyses. This arises from differences in the spread of outbreaks, but also in uncertainty over the proportions of people consulting health services and how these consultations will be coded to a particular syndromic indicator by a health professional [7, 8, 12]. Also, few studies have investigated important information such as whether detection abilities vary according to the size of the outbreak, its duration, time of year or geographical location [7, 12].

Here, we present an evaluation framework used for an exhaustive evaluation of the detection capabilities of syndromic surveillance systems. For this paper, we use England as a case study but the comprehensive evaluation framework could just as easily be applied in different international settings with varying outbreak detection systems. The results are presented for two contrasting public health events: (1) a national pandemic of influenza and (2) a localised outbreak of cryptosporidiosis associated with a public water supply. While the specific results are of most relevance to one country (England) we suggest that many of the broad themes may have wider applicability.

### Context and specific aims

In England, the Real-time Syndromic Surveillance Team (ReSST) at Public Health England (PHE) obtains and analyses data from four National Health Service (NHS) healthcare settings. These systems are based upon NHS-111 (a telehealth consultation system) calls [17], in-hours General Practitioner (GPIHSS) and out-of-hours and unscheduled care General Practitioner (GPOOHSS) consultations [18, 19], and emergency department (ED) attendances (EDSSS) [20]. In England, a General Practitioner is a medical generalist doctor who acts as the first point of contact between patients and the NHS.

Each of the four PHE syndromic surveillance systems uses daily data on syndromic health indicators (e.g. influenza-like illness recorded in GPOOHSS). The health indicators from these syndromic surveillance streams are analysed on a daily basis using epidemiological methods and within a multi-level mixed-effects model (RAMMIE) for the detection of aberrations [21]. Each health indicator from these different surveillance system is routinely aggregated and analysed at national, regional and local levels.

The aims of this study were three-fold. First, we investigated how the characteristics of different disease outbreaks (e.g. outbreak size, time of year and geography of affected area) affected whether an outbreak was detected and the time to detection. Second, we studied the utility of different syndromic surveillance systems (e.g. NHS-111 vs. GPIHSS) to detect different disease outbreaks. Lastly, we investigated the potential effects of an increase in syndromic surveillance coverage (e.g. proportion of emergency departments reporting to ReSST) on time to detection and probability of detection of an outbreak.

## Methods

### Overview of the evaluation framework

To fully assess the detection capabilities of syndromic surveillance systems in England an evaluation framework was developed. This framework builds upon the studies conducted by Morbey et al. [13], Bolt et al. [7], and Jackson et al. [12].

**Fig. 1:**
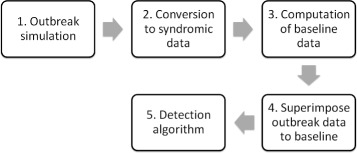
Framework overview. Stages of the proposed framework for the evaluation of aberration detection methods

The proposed framework uses the outbreak detection system implemented in England as a case study and has five main stages (Fig. 1). First, we used compartmental models to simulate epidemic curves (outbreaks) of varying characteristics (e.g. outbreak size, length of the incubation and infectious periods). Second, we converted the simulated outbreak data into syndromic indicator data for different syndromic data streams (e.g. influenza-like illness consultations to GPOOHSS) using a series of scaling factors. Third, for each of these syndromic indicator datasets, baseline data (i.e. expected numbers of reports in the absence of an outbreak) were computed based on historical time series analysis of observed syndromic surveillance data. Fourth, the simulated syndromic indicator data were added to the baseline time series to produce evaluation datasets. Finally, the evaluation datasets were input into the detection system, and the time to detection (*T*_*D*_) and the probability of detection (*P*_*D*_) were calculated for each outbreak simulation.

One novel feature of our work is that uncertainty is explicitly accounted for. Hence, for each simulated outbreak multiple simulations were produced to account for uncertainty (e.g. in outbreak size and the proportion of individuals consulting health care). All these stages are fully described in the following sections.

### Description of scenarios

Two scenarios were used here to exemplify the usefulness of the proposed framework. These were two health threats for which syndromic surveillance systems would be required for providing early warning. The first scenario consisted of a pandemic caused by a novel strain of influenza arriving into England. Influenza cases were assumed to be randomly imported into the country with a mean of five cases per day for a 90-day period. Autochthonous transmission begins after the arrival of the first imported cases. The new strain was assumed to behave similarly to the 2009/2010 A(H1N1)pdm09 ‘swine flu’ pandemic with a rapid spread throughout the population due to lack of immunity [22]. As a pandemic is likely to spread across the whole country, national level syndromic indicators were examined.

The second scenario was a point-source release of *Cryptosporidium spp.* oocysts (the organism responsible for cryptosporidiosis) into a public water supply system. Contamination was assumed to occur at a continuous rate over a three-day period after which public health action (e.g. boil-water advisory) is taken to prevent further intake of contaminated water. Public water supply outbreaks are likely to be geographically restricted (see for example [23]). Hence, the analysis of the syndromic data focused upon sub-national geographies. To further explore the potential influence of geography, the cryptosporidiosis outbreaks were modelled to occur in three randomly chosen locations: one large metropolitan area (location A), and two smaller, predominantly rural areas (locations B and C).

### Step 1: outbreak simulation

Outbreaks of pandemic influenza A(H1N1)pdm09 and cryptosporidiosis were simulated using disease-specific compartmental models. A compartmental model is a series of differential equations that estimate the number of infective people per unit time (e.g. days) based on different parameters (e.g. transmission rate, incubation and infectious periods) of the disease under scope [24]. A full description of the compartmental models can be found in the Additional file 1.

Three outbreak sizes were defined for each disease. For pandemic influenza, outbreak size was defined as a function of the basic reproduction number (*R*_0_) which can be thought as the number of secondary infections generated per primary case. The size levels defined correspond to the 10^*th*^, 50^*th*^ and 90^*th*^ percentiles of the range of *R*_0_ values (i.e. 1.57, 2.25 and 2.93) found on previous studies for pandemic influenza A(H1N1)pdm09 [25–28]. For cryptosporidiosis, outbreak size was defined in terms of the number of people consuming un-boiled contaminated water over a three-day exposure period based on the established literature [23, 29–32] and expert knowledge. Thus, each day over the exposure period, a Poisson-distributed random number of people with mean *λ* is exposed to the contaminated water source. For comparative purposes, the same *λ* values were used for locations A, B and C. Detailed information about the specific values used on each model can be found on the Additional file 1.

All possible combinations of the 10^*th*^, 50^*th*^, and 90^*th*^ percentiles of the range of values for the model parameters indicated on Table 1 were used to simulate numerous possible outbreak signals and to explore uncertainties in our estimates. Data were simulated at daily time steps for coherence with the syndromic data. All simulations were performed in *R* [33] using the *deSolve* package [34].

**Table 1:** Model parameters. Parameters used for the compartmental models of influenza and cryptosporidiosis

Parameter	Range	Reference	Uncertainty explored
Influenza			
Reproductive Number (*R*_0_)	1.4–3.1	[25–28]	Yes
Incubation period (*σ*)	1.0–7.0 days	[26, 46–50]	Yes
Infectious period (*γ*)	2.6–12.0 days	[28, 46, 50]	Yes
Fraction of symptomatic people (*p*)	0.5–0.75	[46, 50]	Yes
Infectivity reduction in asymptomatic people (*k*)	0.1–1.0	[28]	Yes
Cryptosporidiosis			
Number of people exposed to contaminated water per day (*λ*)	747–10354	[23, 29–32]	Yes
Average daily un-boiled water consumption in litres (*w*)	1.8 L	[29]	No
Number of oocysts released into the water system per litre (*o*)	10-1,000,000	[30–32]	Yes
Dose-response hyper-parameters (*α*, *β*)	0.115, 0.176	[31, 51]	No
Incubation period (*σ*)	1.0–21.0 days	[29, 30, 52, 53]	Yes
Infectious period (*γ*)	3.0–50.0 days	[30, 54]	Yes
Fraction of infectious and symptomatic people (*p*)	0.2–0.9	[52, 54]	Yes

### Step 2: conversion to syndromic data

Cases from the outbreak simulations were converted into the numbers of people estimated to consult healthcare systems and hence be captured by one of the four PHE syndromic surveillance data streams. This was achieved by multiplying the compartmental model output by the 10^*th*^, 50^*th*^, and 90^*th*^ percentiles of the range of values of the proportion of the population estimated to consult different forms of healthcare (Table 2) [35, 36]. Not all healthcare providers report syndromic surveillance data to the ReSST, and so simulated data were also multiplied by the estimated coverage of each syndromic system (Table 2). Finally, simulated data were scaled by the estimated proportion of people coded to a syndromic indicator (Table 2). The estimates for the proportion of the population seeking medical advice for the selected conditions are not well known. Therefore, here they were estimated based on previous studies [35, 36] and expert knowledge.

**Table 2:** System features. Percentage of people consulting different healthcare providers, percentage of people coded to an indicator, and proportional coverage of each PHE syndromic surveillance system. Upper and lower estimates are presented in brackets

System	People consulting healthcare (%)		Estimated current coverage (%)				People coded to an indicator (%)	
	Influenza	Cryptosporidiosis	Influenza	Cryptosporidiosis			Influenza	Cryptosporidiosis
	National	All sites	National	Location A	Location B	Location C	National	All sites
EDSSS	0.91% (0.5–5.0%)	0.01% (0.0025–0.023%)	7%	15%	23%	47%	6%	75%
GPIHSS	10% (5.0–30.0%)	2.3% (1.0–5.6%)	64%	64%	95%	86%	6%	75%
GPOOHSS	1% (0.5–3.0%)	2.3% (1.0–5.6%)	65%	65%	7%	33%	29%	75%
NHS-111	1.28% (0.25–10.0%)	0.8% (0.1–0.9%)	100%	100%	100%	100%	22%	75%

In this study we assumed that the probability (*p*) of symptomatic people (*i*) seeking medical attention will vary depending on how many days have passed since the onset of symptoms. Following Fan et al. [6] we assumed the following probability distribution of symptomatic people seeking medical attention: 55.9% on day 1, 35.7% on day 2, 7.2% on day 3, 0.8% on day 4, 0.2% on day 5 and 0.2% on day 6. The same probabilities were used for influenza and cryptosporidiosis. The matrix below illustrates how the number of new outbreak cases *i* at day *t* (denoted by *i*_*t*_) were calculated to seek medical attention based on the probabilities *p*_*t*_. As the matrix shows, at day *t*=1, *p*_1_*i*_1_ people will seek medical attention the same day, *p*_2_*i*_1_ people will seek medical attention the day after, and so on until day six after the onset of symptoms. At day *t*=2, *p*_1_*i*_2_ people will seek medical attention the same day, *p*_2_*i*_2_ will do so the day after, and so on.

$$P_{t}I_{t}= \left[ \begin{array}{cccccc} p_{1}i_{1} & 0 & 0 & 0 & \dots & 0 \\ p_{1}i_{2} & p_{2}i_{1} & 0 & 0 & \dots & 0 \\ p_{1}i_{3} & p_{2}i_{2} & p_{3}i_{1} & 0 & \dots & 0 \\ \vdots & \vdots & \vdots & \vdots & \ddots & \vdots \\ p_{1}i_{t} & p_{2}i_{t-1} & p_{3}i_{t-2} & p_{4}i_{t-3} & \dots & p_{t}i_{t-5} \end{array} \right] $$

### Step 3: computation of baseline data

The most sensitive codes used by clinicians for capturing people with signs and symptoms of pandemic influenza are *cold*/*flu* when accessing NHS-111 [17], and influenza-like-illness via GPIHSS, GPOOHSS [18, 19], or EDSSS [20]. Hence system-specific and indicator-specific baseline data were obtained from the Rising Activity, Multi-level Mixed-effects, Indicator Emphasis (RAMMIE) model [21], which is currently used for routine surveillance in England.

Here, we use RAMMIE as a study case. In other settings different detection methods such as CUSUM or Farrington Flexible could be substituted [37, 38]. This model uses as its input historical long-term records of syndromic data. Briefly, RAMMIE is a multi-level mixed-effects regression model that benefits from the hierarchical structure of the syndromic surveillance signals which are subsets of national signals [21]. A negative binominal functional form is used to account for possible over-dispersion in the syndromic surveillance data. To allow for changes in data volume, the model uses an offset (registered patient population of GP practices or total daily activity depending on the data stream). The model accounts for the effects of independent variables including day of the week, bank holidays, and month of the year. Further details of the RAMMIE model can be found elsewhere [21]. Here, we use RAMMIE to estimate the mean number of system-specific and indicator-specific syndromic counts (henceforth baseline data), and their corresponding detection thresholds. This methodology is different to other well known methods such as cumulative sums (CUSUM) [37], exponentially weighted moving averages (EWMA) [39], or sequential probability ratio tests (SPRT) [40] which are also used in syndromic surveillance.

Baseline data for influenza were aggregated at the national level. We assume that people with signs and symptoms of cryptosporidiosis are most likely to be coded as *diarrhoea* in all four syndromic surveillance systems, and these data were modelled using the RAMMIE model for the same period as the influenza data. However, for cryptosporidiosis baselines were estimated for each of the 3 locations independently.

Syndromic baseline data would typically vary between years. To explore these effects on outbreak detection, we simulated 100 Monte-Carlo samples for each baseline time series using random sampling. Gaussian and Poisson distributions were used to simulate the Monte-Carlo samples. The decision as to whether a Gaussian or a Poisson distribution would be used for a given baseline was based on the distribution of the original data. Thus, if the original time series was Poisson distributed, a Poisson distribution was also assumed for each of the simulated time series. The number of Monte-Carlo samples was governed by the large computation time required to test our scenarios.

### Step 4: superimpose outbreak data to baseline

Evaluation datasets were created by adding the syndromic data to each of the 100 Monte-Carlo simulated baseline time series. The process was repeated varying the start date of outbreaks (on every other day) across the whole year to explore potential effects of month of the year on outbreak detection.

### Step 5: detection algorithm

Given the large number of simulations to be assessed, we would expect some statistical alarms by chance. Hence to reduce the impact of false alarms on *T*_*D*_ and *P*_*D*_, detection is defined as the earliest day after which the evaluation data alarms for three or more consecutive days. We take the pragmatic approach of prioritising exceedances that have repeated for three or more consecutive days in a row because the chance of repeated consecutive false alarms is significantly less likely than single day false alarms. In addition, all the synthetic outbreaks considered here are considerably longer than three days in duration.

Here, *T*_*D*_ was considered as the time elapsed between the onset of the outbreak and the first day of its detection (i.e the first of three days) [12]. The median *T*_*D*_ for an outbreak was computed as the median across the 100 Monte-Carlo-derived evaluation time series for a given baseline. Studentized bootstrap sampling was used to estimate the 95% confidence intervals for the median *T*_*D*_ based on 1000 samples. The ReSST examine all statistical alarms on weekdays using a risk assessment tool [5] and thus, in the event of an alarm which looks particularly unusual from a public health perspective, public health authorities could be highlighted about detection two days earlier than estimated using this framework. The *P*_*D*_ was calculated as the proportion of detected outbreaks across all 100 Monte-Carlo-derived evaluation series [12]. Outbreaks with a *P*_*D*_ lower than or equal 50% were considered as not detected. Median *T*_*D*_ was preferred over mean *T*_*D*_ because the median is more robust to the presence of outliers.

### Estimating what size of an outbreak is required to achieve a 50% probability of detection

Although we simulated a considerable number of outbreaks of different sizes, from a public health point of view it is important to understand the size of outbreaks that can and cannot be detected. Here, we defined detection as a *P*_*D*_ of 50% or greater. Thus, to achieve this, we incrementally increased the size of the outbreaks by increasing the number of people affected by water contaminated with *Cryptosporidium spp.* oocysts until the outbreak was detected. For this exercise, we kept the length of the incubation and infectious periods, the probability of infection, and the proportion of asymptomatic people constant to their 50^*th*^ percentile values. For influenza, outbreak size was increased incrementally by increasing the *R*_0_ until an outbreak was detected. As in the case of cryptosporidiosis, the value of all other parameters remained unchanged.

### Comparing time to detection and probability of detection under a 100% coverage assumption

In an ideal situation, there would be 100% coverage by all syndromic surveillance systems to obtain data for the entire population. This is currently not the case for all systems. We investigated how a hypothetical 100% coverage in each syndromic surveillance system would affect *T*_*D*_ and *P*_*D*_, by scaling up the overall mean on each syndromic time series whilst assuming that the variance in the sample was representative of the entire population. Syndromic data were scaled up as follows:

$$ Y_{i} = x_{i}~-~\mu_{i}~ + ~ \nu_{i} $$

$$ \nu = \mu ~ / ~ coverage $$

where *Y*_*i*_ is the time series of scaled syndromic data estimated for syndromic surveillance system *i*, under the assumption of a 100% coverage, *x* is the observed time series of syndromic data, *μ* is the mean of *x*, and *ν* is the estimated mean of *x* assuming a 100% coverage. The scaled time series were then used as a baseline for the evaluation framework. Simulated outbreak data were then imposed onto the scaled up syndromic baseline data after being converted into numbers of people expected to consult healthcare, and to be coded to each of the indicators considered in the study.

## Results

The EDSSS and GPOOHSS systems had a lower mean number of consultations than the other two systems for the influenza and cryptosporidiosis indicators (see Additional file 1). A total of 4,422,600 time series (i.e. 243 outbreaks ×100 Monte-Carlo-derived baseline time series × 182 initial dates) were simulated for each syndromic indicator for a total of 70,761,600 evaluation time series (4,422,600 time series ×4 locations ×4 indicators per location). Comparisons between historical outbreaks and the simulated epidemic curves for each disease are presented in the Additional file 1 and both show good agreement.

For each scenario, *P*_*D*_, *T*_*D*_, and the numbers of symptomatic people at the detection point are presented in Table 3. In this table, results are presented stratified by indicator and outbreak size, and we recall that three outbreak sizes were defined for each disease and, in the case of cryptosporidiosis, for three different locations. We note that given that detection is defined as three consecutive alarm days the probability of observing a false positive under our scenarios is extremely low.

**Table 3:** Detection metrics. Mean probability of detection (*P*_*D*_), median days to detection (*T*_*D*_) and thousand symptomatic people (Sym) at median time to detection per syndromic surveillance system and outbreak size under the current coverage and a hypothetical 100% coverage for each syndromic surveillance system

		Estimated current coverage									Hypothetical 100% coverage								
		Size 1			Size2			Size 3			Size 1			Size2			Size 3		
System-indicator	Coverage (%)	*P*_*D*_	*T*_*D*_	Sym	*P*_*D*_	*T*_*D*_	Sym	*P*_*D*_	*T*_*D*_	Sym	*P*_*D*_	*T*_*D*_	Sym	*P*_*D*_	*T*_*D*_	Sym	*P*_*D*_	*T*_*D*_	Sym
Influenza																			
EDSSS-influenza like illness	7	1.00	158	105.6	1.00	87	110.9	1.00	64	115.0	1.00	97	7.4	1.00	56	7.8	1.00	42	8.1
			(81–248)			(44–136)			(32–99)			(52–161)			(31–91)		(23–68)		
GPIHSS-influenza like illness	64	1.00	102	9.4	1.00	61	12.6	1.00	47	14.2	1.00	93	6.1	1.00	56	8.1	1.00	43	9.1
			(56–162)			(33–96)			(25–73)			(51–148)			(31–89)			(23–68)	
GPOOHSS-influenza like illness	65	1.00	146	53.4	1.00	81	59.5	1.00	59	63.3	1.00	136	34.7	1.00	76	38.7	1.00	56	41.1
			(74–222)			(41–124)			(30–91)			(70–208)			(39–116)			(29–86)	
NHS-111 cold-flu	100	1.00	140	72.0	1.00	79	79.6	1.00	58	84.0	1.00	140	72.0	1.00	79	79.6	1.00	58	84.0
			(76–232)			(42–128)			(30–94)			(76–232)			(42–128)			(30–94)	
Cryptosporidiosis (Location A)																			
EDSSS diarrhoea	15	0.00	-	-	0.00	-	-	0.00	-	-	0.00	-	-	0.00	-	-	0.00	-	-
GPIHSS diarrhoea	64	0.14	-	-	0.24	-	-	0.84	8	4.4	0.25	-	-	0.39	-	-	0.92	6	3.6
									(2–33)									(2–24)	
GPOOHSS diarrhoea	65	0.18	-	-	0.33	-	-	0.95	4	2.4	0.34	-	-	0.51	7	0.9	0.99	3	1.8
									(2–11)						(3–20)			(1–9)	
NHS-111 diarrhoea	100	0.00	-	-	0.00	-	-	0.52	7	5.7	0.00	-	-	0.00	-	-	0.52	7	5.7
									(3–21)									(3–21)	
Cryptosporidiosis (Location B)																			
EDSSS diarrhoea	23	0.00	-	-	0.00	-	-	0.00	-	-	0.00	-	-	0.00	-	-	0.00	-	-
GPIHSS diarrhoea	95	0.63	10	0.4	0.76	7	0.6	1.00	3	1.4	0.65	9	0.4	0.77	7	0.6	1.00	3	1.4
			(3–47)			(2–37)			(1–7)			(3–47)			(2–36)			(1–7)	
GPOOHSS diarrhoea	7	0.05	-	-	0.12	-	-	0.83	4	3.4	0.93	4	0.2	0.97	3	0.3	1.00	2	0.6
									(2–12)			(2–10)			(2–8)			(1–3)	
NHS-111 diarrhoea	100	0.10	-	-	0.23	-	-	0.89	4	3.2	0.10	-	-	0.23	-	-	0.89	4	3.2
									(2–13)									(2–13)	
Cryptosporidiosis (Location C)																			
EDSSS diarrhoea	47	0.00	-	-	0.00	-	-	0.00	-	-	0.00	-	-	0.00	-	-	0.00	-	-
GPIHSS diarrhoea	86	0.62	12	0.4	0.74	9	0.6	0.99	3	1.7	0.67	11	0.4	0.78	9	0.6	1.00	3	1.5
			(3–53)			(3–47)			(1–9)			(3–51)			(3–41)			(1–8)	
GPOOHSS diarrhoea	33	0.44	-	-	0.61	6	0.8	0.99	3	1.7	0.85	5	0.4	0.94	4	0.4	1.00	2	0.8
						(3–17)			(1–8)			(2–13)			(2–11)			(1–4)	
NHS-111 diarrhoea	100	0.06	-	-	0.18	-	-	0.86	4	3.3	0.06	-	-	0.18	-	-	0.86	4	3.3
									(2–14)									(2–14)	

*T*_*D*_ and number of symptomatic people for *P*_*D*_≤0.5 are not presented

The values in brackets indicate the 95% prediction intervals for *T*_*D*_

All outbreaks of pandemic influenza were detected by every syndromic surveillance system (*P*_*D*_= 1.00). The median false positive rate accross systems was zero. There were statistically significant between-system differences (ANOVA, *p* <0.001) in median *T*_*D*_ for influenza. We noted that *T*_*D*_ was negatively related to the size of the outbreak. Thus, for example, the GPIHSS-influenza-like-illness indicator, the influenza-related indicator with the lowest *T*_*D*_ (Table 3), detected outbreaks of size 1 at day 102 (95% C.I. 56–162 days), outbreaks of size 2 at day 61 (95% C.I. 33–96 days), and outbreaks of size 3 at day 47 (95% C.I. 25–73 days). At these times in the outbreak we would expect about 9447, 12612, and 14201 cases of symptomatic pandemic influenza in England respectively. After GPIHSS, detection is followed by NHS-111-cold/flu, GPOOHSS-influenza-like-illness and EDSSS-influenza-like-illness.

Conversely, Table 3 indicates that the *P*_*D*_ for outbreaks of cryptosporidiosis was highly variable ranging between 0–100%. This situation highlights the advantages of incorporating uncertainty into the analysis. The false positive rate was zero for all three locations. Using a detection threshold of 50%, almost none of the outbreaks of size 1 were detected by any of the system-indicator combinations in any of the three areas. The exception was GPIHSS-diarrhoea that was able to detect approximately 60% of the outbreaks of size 1 but only in Locations B and C. The *P*_*D*_ became closer to one as the size of the simulated outbreaks increased. The size 2 outbreaks were only detected in the locations B and C and not by all systems whereas outbreaks of size 3 where detected in all three locations and by all syndromic surveillance systems except for EDSSS. In terms of efficacy, the GPIHSS-diarrhoea and GPOOHSS-diarrhoea were the most sensitive for the detection of outbreaks of cryptosporidiosis with GPIHSS being slightly more sensitive. However, the most efficient indicator varied between areas. For example, in Location A GPOOHSS had the highest *P*_*D*_ for a size 2 outbreak whereas in Location B GPIHSS had the highest *P*_*D*_. NHS-111-diarrhoea detected very few outbreaks but EDSSS-diarrhoea detected none. There were statistically significant differences in *P*_*D*_ between syndromic indicators for the two diseases (ANOVA, *p* <0.001).

Due to the point source nature of the outbreaks of cryptosporidiosis where many individuals are exposed to the pathogen over a brief period of time, *T*_*D*_ was considerably lower than that for influenza (Table 3). Statistically significant differences (ANOVA, *p* < 0.001) were observed in median *T*_*D*_ across indicators for cryptosporidiosis in the three areas. Based on the results of the most sensitive syndromic surveillance system (GPIHSS and GPOOHSS), the median *T*_*D*_ for oubreaks of cryptosporidiosis of size 3 was three days except for location A where it was 4 days.

This variability in *P*_*D*_ and *T*_*D*_ lead to variations in the size of outbreak that can be detected (i.e. the numbers of symptomatic people at the point of detection). For example, if we focus upon a size 3 outbreak of cryptosporidiosis then Table 3 indicates that at the point of detection there would be between 1400 symptomatic individuals in Location B, 1700 in Location C, and 4400 in location A. GPIHSS-diarrhoea and GPOOHSS-diarrhoea appeared similar in their detection ability with the best system varying between the three areas.

**Fig. 2:**
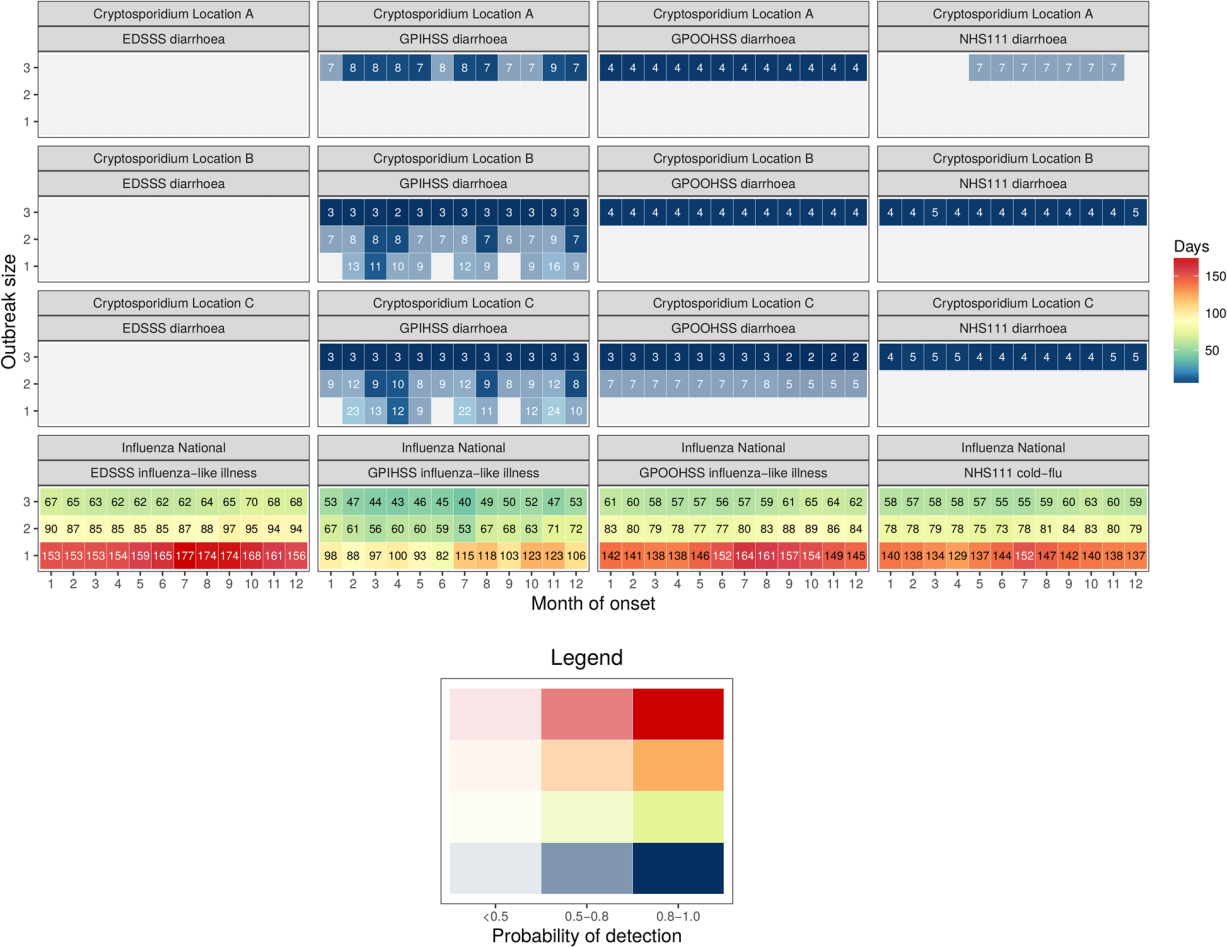
Median time to detection per month. Median *T*_*D*_ for influenza and *Cryptosporidium spp.* outbreaks stratified by month of the onset and syndromic indicator. The colour of the boxes indicate the median *T*_*D*_. The darkness of the box filling indicates the probability of detection (*P*_*D*_) as defined in the legend. Boxes with a *P*_*D*_≤0.50 were excluded

Figure 2 compares the median *T*_*D*_ for each system stratified by month, and indicates that for influenza, *T*_*D*_ depends upon when the first case occurs. An outbreak with a first influenza case with onset between February and July tended to have, on average, a lower *T*_*D*_ in comparison to an initial case with onset between August and January. For example, an outbreak of influenza of size 3 starting in July is detected, on average, seven days earlier than one starting in November (e.g. 40 vs. 47 days based on GPIHSS influenza-like-illness). Conversely, for cryptosporidiosis Fig. 2 indicates few clear patterns suggesting little seasonal variation on *T*_*D*_.

We then used our framework to investigate the minimum size an influenza or cryptosporidiosis outbreak that would be detected at least 50% of the time. The results indicate that the number of symptomatic people at the median *T*_*D*_ required to achieve a *P*_*D*_ of approximately 0.5 varies considerably between systems (Table 4). For influenza, the smallest detectable outbreak was estimated to have an *R*_0_ of 1.09 secondary cases per primary case. Such an outbreak would be detected by the GPIHSS system in about 358 days as the number of symptomatic people reaches three thousand. We noted that compared to the other three systems, the number of symptomatic people required to obtain a *P*_*D*_ of about 0.5 was an order of magnitude lower for the GPIHSS system (three thousand compared against 48–82 thousand). This results concur with the results in Table 3.

**Table 4:** Outbreak size required for *P*_*D*_ of 0.5

Influenza				
System	Coverage	Secondary cases	*T*_*D*_ (days)	Symptomatic (thousand)
EDSSS	15%	1.26	318	81.9
GPIHSS	64%	1.09	358	3.0
GPOOHSS	65%	1.22	332	47.7
NHS-111	100%	1.22	348	63.1
Cryptosporidiosis				
System	Coverage	People exposed (thousand)	*T*_*D*_ (days)	Symptomatic (thousand)
(Location A)				
EDSSS	15%	1,750.0	13	492.7
GPIHSS	64%	11.3	22	3.3
GPOOHSS	65%	4.2	18	1.3
NHS-111	100%	12.5	18	3.8
(Location B)				
EDSSS	23%	512.4	13	144.4
GPIHSS	95%	2.2	19	0.7
GPOOHSS	7%	7.8	17	2.3
NHS-111	100%	3.5	16	1.0
(Location C)				
EDSSS	47%	284.6	17	84.9
GPIHSS	86%	2.8	21	0.9
GPOOHSS	33%	2.4	18	0.7
NHS-111	100%	4.1	18	1.3

Estimated size of influenza and *Cryptosporidium spp.* outbreaks required to achieve at least a 0.5 probability of detection per syndromic surveillance system and region

For cryptosporidiosis, the estimated minimum number of exposed people required for achieving about 0.50 *P*_*D*_ ranged between 2200 and 4200 people depending on the location under scope. This equates to 700 to 1300 people experiencing symptoms. Thus, in location A, the minimum number of exposed people required for about 0.50 *P*_*D*_ was 4200. Such an outbreak would be detected in approximately 18 days as the number of symptomatic people reaches 1300. In locations B and C, however, the minimum size of the outbreak required for about 0.50 *P*_*D*_, was considerably lower (i.e. 2200 and 2300 exposed people, respectively) although *T*_*D*_ was similar (18–19 days). As expected, given the low *P*_*D*_ observed in the EDSSS-diarrhoea indicator, the estimated exposed population required to achieve a about 0.50 *P*_*D*_ was considerably greater for the EDSSS system than for the others.

We then investigated the effects of an increase in system coverage to a 100% on each syndromic surveillance system (Table 3). We noted a consistent decrease in *T*_*D*_ in most indicators compared to their current system coverage, and the greatest changes occurred unsurprisingly in the system-indicator combinations where coverage was lowest. For influenza, the biggest change in *T*_*D*_ was observed in the EDSSS-influenza-like-illness indicator where we estimate an average reduction of approximately 36% in *T*_*D*_ compared to its current coverage. From a public health perspective EDSSS-influenza-like-illness then has a *P*_*D*_ and *T*_*D*_ comparable to the best system-indicator combinations and is now clearly more sensitive than NHS-111-cold/flu. Should all the indicators achieve a 100% coverage then the best *T*_*D*_ falls to 93, 56, and 42 days for a size 1, 2 and 3 outbreak respectively.

For cryptosporidiosis, the biggest relative decrease in *T*_*D*_ (50%) was observed in location B for the GPOOHSS-diarrhoea indicator which changed from four to two days for outbreaks of size 3; an unsurprising result as the existing coverage is 7%. It is noted that similar absolute changes in *T*_*D*_ (2 days) were observed in other locations and indicators such as GPIHSS-diarrhoea in location A for outbreaks of size 3 (8 to 6 days), and GPOOHSS-diarrhoea for outbreaks of size 2 (6 to 4 days). The increase in coverage also had an effect on *P*_*D*_. Thus, for example, in location B, *P*_*D*_ for the GPOOHSS-diarrhoea indicator increased from 5 to 93% for outbreaks of size 1, from 12 to 97% for outbreaks of size 2, and from 83 to 100% for outbreaks of size 3. Should all the indicators achieve a 100% coverage then the best *T*_*D*_ falls to 5, 3 and 2 days for a size 1, 2 and 3 outbreak respectively.

**Fig. 3:**
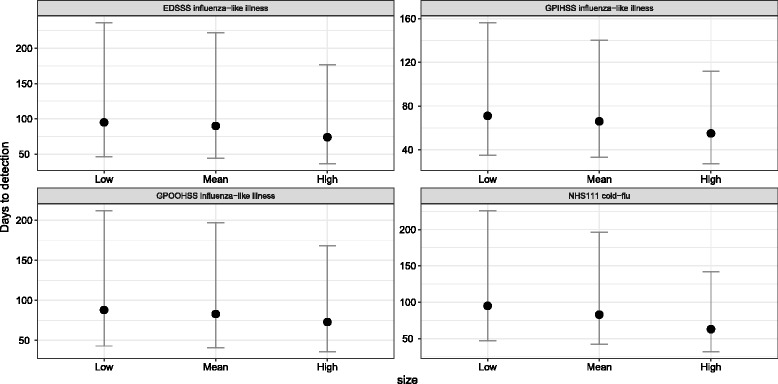
Median *T*_*D*_ per level of access to healthcare. Median *T*_*D*_ for influenza outbreaks across four syndromic indicators for three different levels of access to healthcare (see Table 2). The dots indicate the median *T*_*D*_, and the error bars depict the 95% studentized bootstrap prediction intervals. Prediction intervals were estimated using 1000 bootstrap samples. Estimates with a probability of detection ≤75% were excluded

Our framework allows the investigation of the effects of different proportions of consulting to health services on *T*_*D*_. A negative association was observed between the proportion of people consulting a particular system and *T*_*D*_. Thus, as the proportion of people consulting healthcare services increased, *T*_*D*_ decreased. This situation is shown in Fig. 3 for the four pandemic influenza indicators. As can be observed, the trend was similar across all syndromic surveillance systems. The difference between the three levels of people consulting a healthcare system was not significant (*p* >0.05) using an ANOVA test.

## Discussion

Syndromic surveillance is increasingly used for the timely detection of public health threats supporting existing public health surveillance programmes. The aim is that faster detection will lead to more effective public health action. However, we argue in the introduction that there is a lack of research critically assessing the types of events that can be detected, how long such systems take to detect the event, or characterising those events that cannot be detected.

Here, we present a comprehensive framework for the evaluation of syndromic surveillance systems. Building upon previous work (see for example [7, 12, 13]) it incorporates a large number of different system-syndrome indicators and fully incorporates uncertainty into the assessments. We also explore a wide range of measures relevant to public health such as the probability of and time to detection for outbreaks of a pre-determined size, the minimum size of outbreaks that would be detected, and the numbers of symptomatic individuals at this point. We also explore the utility of various system-syndrome indicators for different diseases, as well as the impact of outbreak location and time of year upon detection. Finally, we model the impact of increasing the proportion of centres reporting data upon our detection metrics. In our view, these methods provide guidance on how syndromic surveillance should be assessed, and demonstrate the benefits of such a thorough assessment.

For England, our results indicate that a national pandemic of influenza similar to the 2009/2010 A(H1N1)pdm09 ‘swine flu’ pandemic with an *R*_0_ of 1.57, 2.25 and 2.93 is likely to be detected at day 102, 61, and 47 respectively at a point in time when there are likely to be 9400, 12600 and 14200 symptomatic individuals. The GPIHSS-influenza-like-illness was the most sensitive indicator. We also estimate that should system coverage increase to 100% the *T*_*D*_ for these three influenza pandemics would fall to 91, 56 or 43 days, respectively. At 100% coverage the EDSSS-influenza like illness indicator performs comparably to GPIHSS-influenza like illness.

Outbreaks of cryptosporidiosis will be more local in nature and the ability to detect outbreaks of different sizes varies by indicator. Small and medium size outbreaks (i.e. about 854 and about 1281 exposed people per day) are not consistently detected in all locations. The largest outbreak (i.e. approximately 8539 exposed people per day), however, is consistently detected but the most sensitive system varies between GPOOHSS and GPIHSS. Detection occurs between 3 and 4 days at a point where there are between 1400 and 2400 symptomatic individuals. The minimum size of outbreak that can be detected varies between 700 and 1300 symptomatic individuals at *T*_*D*_. However, if it takes between 18 and 21 days to detect these smaller outbreaks, the public health utility of these detections is questionable.

We highlight that an increase in coverage of syndromic data streams could improve the systems’ ability to detect outbreaks of cryptosporidiosis at times and locations they would currently go unnoticed. Previous studies using syndromic surveillance data from a range of European likely to be detected at day suggest that the detection of outbreaks of gastrointestinal illness could be challenging, particularly when using emergency department data [41, 42]. Our results support this notion and indicate that even under the assumption of a 100% coverage, the EDSSS-diarrhoea indicator is unable to detect outbreaks that would be detected using the other three systems.

Although these results are specific to the syndromic surveillance system in England, many of our findings are likely to have wider applicability. Firstly, the framework presented in this paper is inherently flexible and enables one to simulate outbreaks of different sizes by allowing the user to modify the parameters that determine their occurrence (e.g. the incubation and infectious periods, and the number of secondary infections per primary case). Our results indicate that *P*_*D*_ increases and *T*_*D*_ decreases as the size of the outbreaks increases, a situation previously observed [12]. Larger outbreaks cause a greater relative increase in case counts when imposed onto the same baseline data than small outbreaks. Also, larger outbreaks tend to peak faster than smaller outbreaks. Previous research on how the size of an outbreak influences *P*_*D*_ and *T*_*D*_ has been constrained to a pre-defined set of values on outbreak duration and size [12, 14, 15]. In our framework the size and duration of an outbreak is determined by the model parameters and so, they could potentially take on any value. We also worked our model “backwards” to identify the minimum size of outbreaks which can be detected.

Most previous research has evaluated the performance of syndromic surveillance using one or two data streams [7, 8, 10–12]. Only a few studies have used multi-stream data for evaluation [6, 13]. Here, we used data from four different syndromic surveillance systems and three different syndromes to explore the uncertainties affecting detection. We highlight the value of using different system-syndrome indicators for event detection. For example, syndromic surveillance data from emergency departments in England are useful for the detection of pandemic influenza but not for the identification of local outbreaks of cryptosporidiosis. This is likely due to the the very low proportion of people expected to attend emergency departments for milder illnesses such as cryptosporidiosis, and the lower proportion of emergency department reporting data [41] (see Table 2). Thus, when outbreak data are scaled down for the EDSSS system, the result is a considerably lower number of symptomatic people being added to the baseline posing difficulties for outbreak detection [12]. Interestingly, emergency department data are the most widely used source of syndromic surveillance data worldwide [41, 43–45]. A further reason why different indicators may be useful for aberration detection may be the specificity of the coding systems used by different syndromic surveillance systems. Thus our results reinforce previous studies (e.g. [6]) highlighting the value of using multiple data streams for improving outbreak detection across diseases.

Our results highlight further reasons why multiple data streams may be useful, as indicator performance may vary geographically. We demonstrate, for the first time, that *P*_*D*_ may vary between geographical areas even when using identical indicators for aberration detection. For example, the ability of NHS-111-diarrhoea, to detect outbreaks of cryptosporidiosis varied between 4 and 7 days between the three locations. These differences emanate from the fact that most detection methods are based upon the prediction interval around a mean value. This interval is based upon historical data and is likely to vary between areas due to factors such as the size of the area, the health seeking behaviour of the population as well as the underlying level of ill health that causes people to seek medical attention. Hence *P*_*D*_ and *T*_*D*_ vary geographically.

To our knowledge, we also demonstrate for the first time that detection ability varies seasonally. For example, an influenza outbreak starting in England in the first half of the year is likely to be detected in a more timely manner than one starting in the second half of the year. This is because the numbers of people reporting different syndromes will vary over the year affecting the prediction intervals and hence the additional level of activity to trigger a statistical alarm.

One advantage of our approach is the incorporation of uncertainty in the computation of aberration detection metrics. Incorporating uncertainty provides a probabilistic assessment of detection which matches the uncertainty in the underlying assumptions. This additionally helps us to understand where the largest uncertainties reside. In England, the proportion of people with an illness who will consult each of the available healthcare services is possibly the largest source of uncertainty. A further source of uncertainty is how these individuals, who have accessed healthcare will be coded to a specific syndrome by healthcare professionals. There are statistically significant differences in *T*_*D*_ for different estimates of people accessing healthcare systems in England. Further research needs to address both these research gaps, and could also provide valuable information for emergency preparedness and response [13]. Our framework offers a useful tool for incorporating these sources of uncertainty in a systematic manner.

One alternative to the use of compartmental models within our framework would be to examine whether actual outbreaks were detected using syndromic surveillance. Potentially, such information could be used to estimate the size of outbreaks that could be detected and the *T*_*D*_. While an important complementary approach, the use of actual outbreaks has its own limitations. All outbreaks have different characteristics, hence comparability between outbreaks is an important issue. Furthermore, a modelling approach enables variables to be changed and their impact examined. We explored how changes to system coverage might influence detection and this could be extended to examine changes to the coding system used within healthcare or the proportion of people seeking healthcare. These are only possible using a modelling framework.

Finally, we re-iterate that although the statistical detection of aberrations in healthcare reporting is important, it is only the first step within the syndromic surveillance process. Following such a detection a complex risk assessment process will usually be used [5] to identify whether public health action is necessary.

## Conclusions

There is little research critically assessing the types of events that syndromic surveillance systems can detect. Here we overcome these limitations through the implementation of a framework for the evaluation of such systems. This framework allows the *T*_*D*_ and *P*_*D*_ for a range of outbreaks to be calculated while fully incorporating uncertainty into the assessment. We demonstrate how such a framework can also model the benefits of increases in the number of centres reporting syndromic data and determine the minimum size of outbreaks that can or cannot be detected. In this paper, the syndromic surveillance systems in England are used as a case study, but our method could easily be applied to varying international settings with varying detection systems. Although the specific results are most pertinent to England, they highlight that small gastrointestinal outbreaks (e.g. cryptosporidiosis) are unlikely to be detected unless the number of symptomatic individuals is over 1000. We also demonstrate the advantages of having multiple syndromic data streams; we show emergency departments data are useful for the detection of pandemic influenza but not for the identification of local outbreaks of cryptosporidiosis. We also highlight that for any one disease, the utility of data streams may vary between different geographical areas, and that the detection ability of syndromic surveillance varies seasonally. For example, an influenza outbreak starting in England in the first half of the year should be detected sooner than one starting in the second half of the year. We argue that our framework constitutes a useful tool for public health emergency preparedness in multiple settings.

## Additional file

Additional file 1 Supplementary Material. Overview of the compartmental models, comparison against real outbreak data, modelling assumptions and residual autocorrelation. (PDF 1800 kb)

## Abbreviations

EDSSS: Emergency Department syndromic surveillance system
GPIHSS: In-hours general practitioner syndromic surveillance system
GPOOHSS: Out-of-hours hours general practitioner syndromic surveillance system
NHS: National Health Service
PHE: Public Health England
*P*_*D*_: Probability of detection
RAMMIE: Rising activity, multi-level mixed effects, indicator emphasis method for syndromic surveillance
ReSST: Real-time syndromic surveillance team
*T*_*D*_: Time to detection
