# Supplementary material for: A methodological framework for the evaluation of syndromic surveillance systems: a case study of England
Source: BMC Public Health. 2018 Apr 24;18:544. doi: 10.1186/s12889-018-5422-9 (PMC5921418; doi:10.1186/s12889-018-5422-9)
Supplement: Supplementary file 1 — Supplementary Material. Overview of the compartmental models, comparison against real outbreak data, modelling assumptions and residual autocorrelation. (PDF 1800 kb) [file 12889_2018_5422_MOESM1_ESM.pdf]

A Methodological Framework for the Evaluation of Syndromic Surveillance Systems: A Case Study of England

Felipe J Colón-González, Iain R Lake, Roger A Morbey, Alex J Elliot, Richard Pebody, Gillian E Smith

Overview of the compartmental models

A compartmental model is formed by a series of differential equations that estimate the number of infectious people per unit time (e.g. days) based on a range of parameters such as the transmission rate, the incubation and infectious periods of a disease [1]. In such kind of models, the individuals in a population are subdivided into categories or "compartments" for which the model tracks the course of the infection process collectively [1]. Compartmental models use differential equations to describe changes in the course of infection in continuous rather than discrete time intervals. Figure 1 depicts the structure of the two compartmental models used in the study. The direction of the solid arrows indicates the flow of individuals from a compartment to another. The dashed arrows depict the contact between an infectious source (e.g. water or an infectious individual) and susceptible individuals. Red colours indicate the presence of an exogenous source of infection.

Influenza model:

Here, we used a compartmental model to simulate the behaviour of a new strain assumed to behave similarly to the 2009/2010 A(H1N1)pdm09 'swine flu'. The model assumes that Poisson distributed infectious individuals (Imp) randomly arrive into England with a mean λ of five cases per day over a 90-day period. Autochthonous transmission begins after the arrival of the first imported cases. Susceptible individuals (S) are then infected at a rate β (the per capita rate at which two individuals come into effective contact [1]) after contact with an infectious person

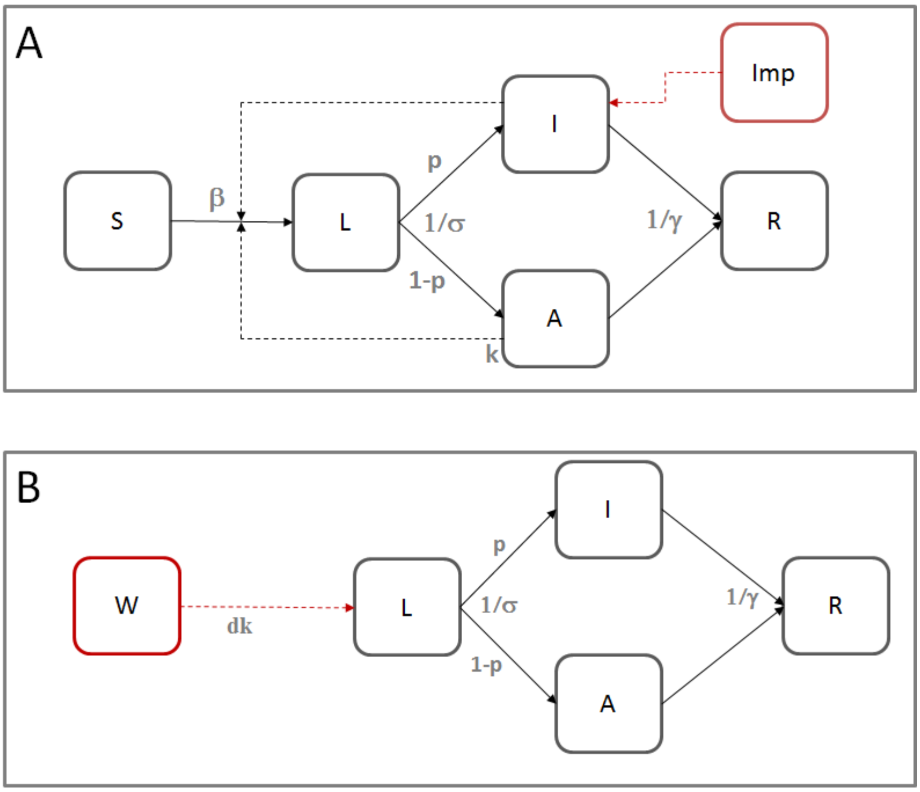

Figure S1: Schematic representation of the compartmental models used in the study. The model in the top (A) was used to simulate outbreaks of pandemic influenza. The model in the bottom (B) was used to simulate outbreaks of cryptosporidiosis.

irrespectively of whether that infectious person is symptomatic (I) or asymptomatic (A). Once infected, individuals become latent (L) carriers of the disease. In latent carriers (L), the disease incubates for a period of σ days. A proportion (p) of latent individuals becomes infectious and symptomatic (I) at a rate of 1/σ per day (1/length of the incubation period). The remainder (1-p) become infectious but asymptomatic (A) also at a rate of 1/σ per day. Asymptomatic individuals have their infectivity reduced by a factor (k). Infectious individuals both symptomatic and asymptomatic, recover (R) at an average rate of 1/γ per day, where γ represents the length of the infectious period (in days). The equations used for pandemic influenza model were as follows [2]:

$$\frac{dS}{dt} = -\beta S(I + kA)$$

$$\frac{dL}{dt} = \beta S(I + kA) - \sigma L$$

$$\frac{dI}{dt} = p\sigma L - \gamma I$$

$$\frac{dA}{dt} = (1-p)\sigma L - \gamma A$$

$$\frac{dR}{dt} = \gamma(I + A)$$

where:

$$\beta = \frac{R_0}{N_0}\left[\frac{p + k(1-p)}{\gamma}\right]$$

$$R_0 = \beta N_0 \left[\frac{p + k(1-p)}{\gamma}\right]$$

$$N_0 = Total\ population$$

Cryptosporidiosis model:

For cryptosporidiosis, we assumed that a Poisson distributed random number of people with mean λ of the population gets infected every day for a 3-day period after drinking k litres of unboiled water from a contaminated source (W). The probability of infection (i.e. the probability that the oocysts can cause infection after being ingested) is represented by d. Once infected, individuals become latent carriers of the disease (L). In latent carriers (L), the disease incubates for a period of σ days. A proportion (p) of latent individuals becomes infectious and symptomatic (I) at a rate of 1/σ per day whilst the remainder (1-p) become infectious but asymptomatic (A) also at a rate of 1/σ per day. Infectious individuals both symptomatic and asymptomatic, recover (R) at an average rate of 1/γ per day, where γ is the length of the infectious period. The differential equations used in this model are given by:

$$\frac{dL}{dt} = -\sigma L$$

$$\frac{dI}{dt} = p\sigma L - \gamma I$$

$$\frac{dA}{dt} = (1-p)\sigma L - \gamma A$$

$$\frac{dR}{dt} = \gamma(I + A)$$

Modelling assumptions

The proposed models have the following shared assumptions:

1. There are no changes in the population size. We make this assumption because it is unlikely to experience significant changes in population size over the short period of the simulations.
2. Individuals are infected at random, and so all susceptible individuals have the same probability of infection when in contact with an infectious source.
3. Once infected, individuals become infectious and recover at a constant rate. This assumption is convenient because when something typically occurs at a constant rate, such rate could be calculated as 1/average time of the event. Thus, for example, if the average incubation period of the parasite is four days (σ = 4 days), the rate at which individuals become infectious equals 1/σ = 1/4 = 0.25 per day (see Figure S1).
4. Recovered individuals remain immune for the whole simulation period. This situation is likely because immunity loss would typically take a period greater than that of our simulations (maximum 365 days).

Cryptosporidiosis-specific assumptions:

Besides the shared assumptions, the model developed to simulate outbreaks of cryptosporidiosis had the following specific assumptions:

1. Infection occurs over a 3-day period after which the water supply is fully decontaminated or temporarily closed.

2. The concentration of oocysts in water remains constant over a 3-day period after which the water supply is fully decontaminated or temporarily closed.

3. The probability of infection due to contact with the contaminated water source depends on both the dose (number of oocysts per daily water intake) and the probability that a single oocyst in the inoculum successfully passes all the host's barriers to infection. The probability of infection is calculated using a hypergeometric (Beta Poisson) dose-response relation [3, 4].

4. There is no shedding of oocysts from the infectious people into the drinking water system.

5. There are no secondary infections due to person-person contact.

6. All oocysts in the water source are viable and infective.

Outbreak size

As specified in the main text, three outbreak sizes were defined for each disease. For pandemic influenza, we defined outbreak size as a function of the basic reproduction number (R_0) which indicates the number of secondary infections expected for each primary case. The levels corresponding to the 10th, 50th and 90th percentiles of the range of R_0 values (1.4–3.1) are presented on table 1 and are based on the established literature for pandemic influenza A(H1N1)pdm09 [5, 6, 7, 8].

Table 1: Interpretation of the three outbreak sizes defined for each of the two diseases considered in the study.

Outbreak size	Interpretation	
	Influenza (R_0)	Cryptosporidiosis (λ)
1	1.57 Secondary infections per primary case	854 people exposed each day for 3 days
2	2.25 Secondary infections per primary case	1281 people exposed each day for 3 days
3	2.93 Secondary infections per primary case	8539 people exposed each day for 3 days

For cryptosporidiosis, on the other hand, outbreak size was defined as a function of the number of people consuming un-boiled contaminated water over the exposure period based on previous studies and expert knowledge [3, 9, 10, 11, 12]. A Poisson-distributed random number of people with mean λ was assumed to get exposed each day to the contaminated water source. Table 1 presents the three values of λ used in the study.

Summary statistics

The table below presents the summary statistics for each of the syndromic surveillance indicators used in the study.

Table 2: Summary statistics of the modelled syndromic baseline time series (cases day^{-1}) used in the study stratified by syndromic surveillance system and indicator.

System	Indicator	Mean	Median	SD	Range
	Influenza				
EDSSS	Influenza-like illness	2.1	1.1	1.8	0.5–10.3
GPIHSS	Influenza-like illness	280.9	164.3	304.8	0.4–1157.4
GPOOHSS	Influenza-like illness	50.7	31.5	54.3	7.3–350.0
NHS-111	Cold/flu	183.4	144.9	130.6	39.8–995.0
	Cryptosporidium (Location A)				
EDSSS	Diarrhoea	4.1	4.0	0.6	2.9–5.9
GPIHSS	Diarrhoea	287.0	377.5	195.4	0.5–599.5
GPOOHSS	Diarrhoea	31.8	22.7	16.0	16.8–77.4
NHS-111	Diarrhoea	75.9	62.3	30.9	39.1–190.1
	Cryptosporidium (Location B)				
EDSSS	Diarrhoea	0.8	0.8	0.1	0.4–1.3
GPIHSS	Diarrhoea	14.4	18.7	9.7	0.1–29.4
GPOOHSS	Diarrhoea	0.4	0.2	0.4	0.1–1.5
NHS-111	Diarrhoea	9.0	5.4	6.1	3.3–24.6
	Cryptosporidium (Location C)				
EDSSS	Diarrhoea	<0.1	<0.1	<0.1	0.0–<0.1
GPIHSS	Diarrhoea	21.2	27.3	14.8	0.1–47.3
GPOOHSS	Diarrhoea	3.5	1.9	4.4	0.1–15.1
NHS-111	Diarrhoea	10.6	7.0	6.2	4.7–26.9

Comparing observed vs. simulated outbreaks

Figure S1 shows the behaviour of the 243 outbreaks of cryptosporidiosis and pandemic influenza used in this study. Simulated outbreak data are represented by the gray lines, and are presented here scaled by the proportion of people estimated to consult the GPIHSS syndromic surveillance system, the proportional coverage of such syndromic surveillance system for location A (cryptosporidiosis) and the whole of England (influenza), and the percentage of people coded with either the diarrhoea or influenza-like illness indicators. For comparative purposes, we included historic data (in red) about outbreaks of both diseases that occurred in the United Kingdom in recent times [13, 14]. As can be observed, the range of simulated data covers the time span and magnitude of the historic outbreak data.

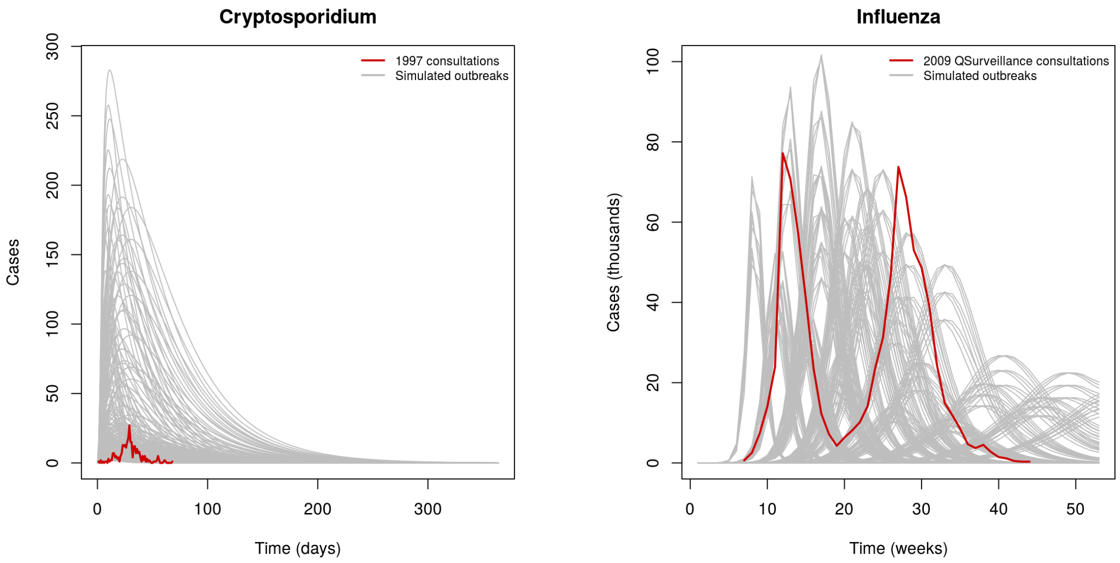

Figure S2: Simulated number of consultations for diarrhoea and influenza-like-illness (gray lines) for the GPIHSS system. Historic observed outbreaks (red lines) are presented as a reference.

Several of the simulated *Cryptosporidium spp.* outbreaks shown on Figure S2 were considerably larger than the historical outbreak whilst most of the simulated pandemic influenza outbreaks were smaller than their corresponding historical data. Figure S2 however, demonstrates that once simulated outbreaks of size 3 are converted to syndromic data, the increase in the expected number of people infected with cryptosporidiosis is rather small and so, some outbreaks may go unnoticed by the detection algorithm. Conversely, for pandemic influenza the expected number of extra cases due to the simulated outbreaks is considerably larger than the baseline and

outbreaks are likely to be easily detected.

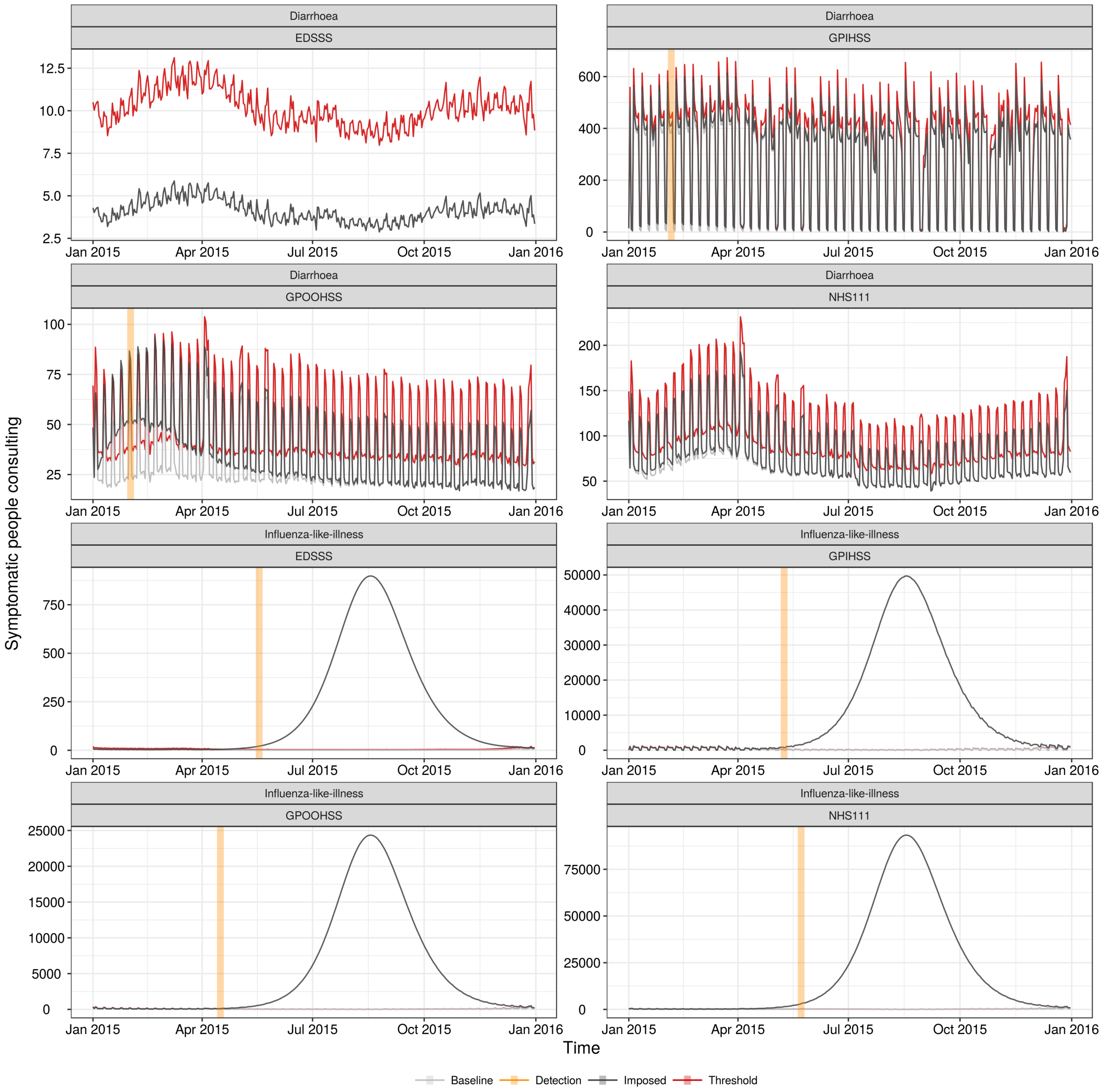

Figure S3: Graphical representation of the evaluation algorithm for outbreaks of size 3 occurring in a metropolitan area (cryptosporidiosis) or across the whole of England (influenza) with onset on 1 January 2015, stratified by disease and syndromic indicator. The light gray lines indicate the baseline syndromic surveillance data; the dark gray lines depict an average evaluation time series (i.e. imposed outbreak data); the red lines represent the statistical alarm threshold estimated with the RAMMIE model; and the vertical thick orange lines indicate the median time to detection.

Alarm Thresholds

The detection algorithm used as an exemplar in this paper is RAMMIE, and full details of this model are provided elsewhere [15]. Every day RAMMIE analyses more than 12,000 separate time series. The key output from RAMMIE are predictions of the mean number of system-specific and

indicator-specific syndromic counts (henceforth baseline data), and their corresponding alarm thresholds (around 99% prediction intervals). These thresholds are used in the operational public health system as a very conservative estimate of potentially unusual activity. In considering the alarm thresholds generated by RAMMIE potential autocorrelation in the residuals were explored. Figure S1 presents autocorrelation plots for the 14 raw data streams input into RAMMIE which were used to generate the baselines and alarm thresholds for this paper.

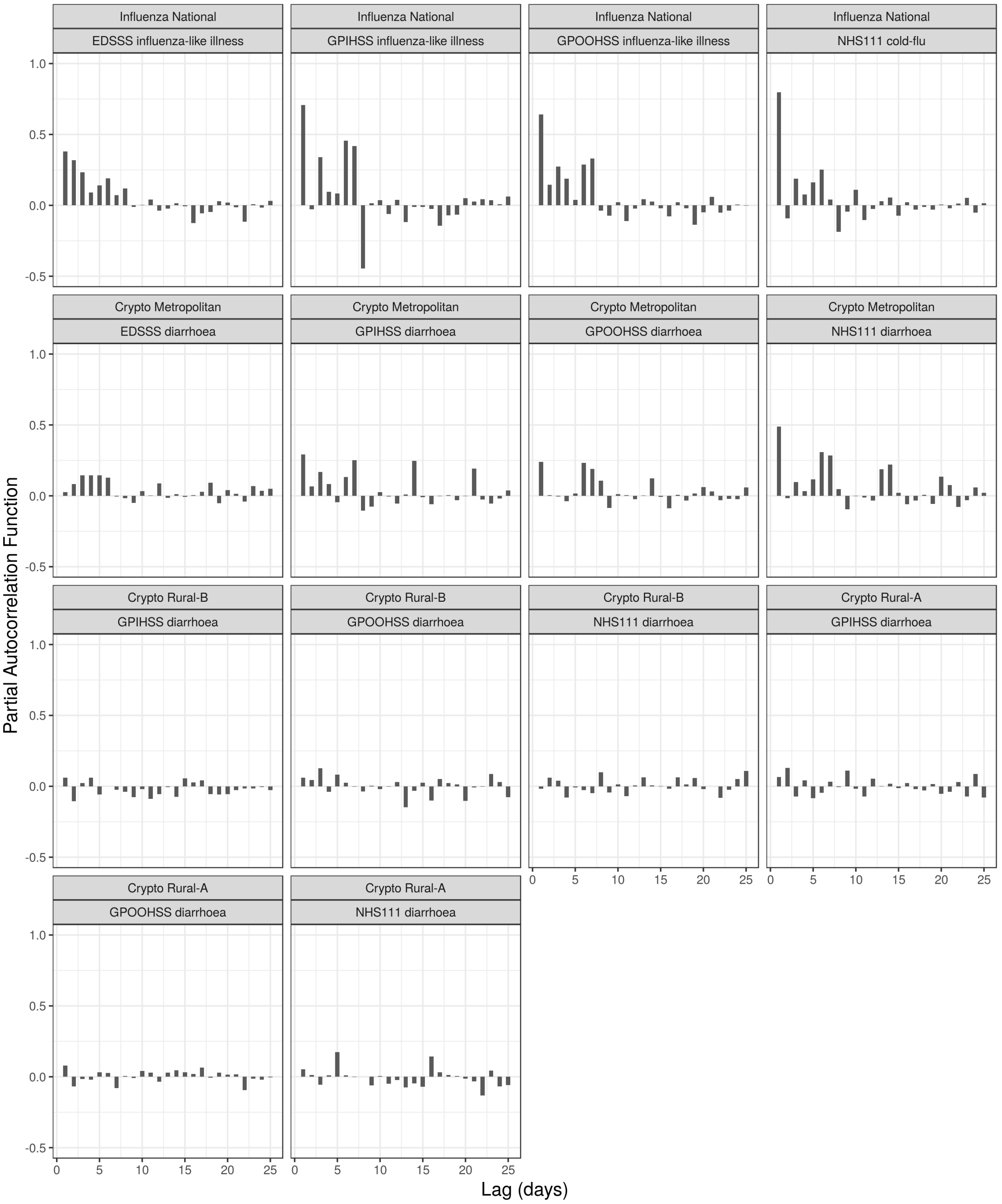

Figure S4: Partial autocorrelation plots for 14 RAMMIE-derived residuals from the datasets used to generate the baseline data and their corresponding alarm thresholds.

Overall, the mean temporal autocorrelation at 1 day lag from these 14 data streams is moderate at around 0.3. However, there is variation between data stream, and lower autocorrelations at day 1 are apparent in the data streams for *Cryptosporidium* as opposed to influenza-like illness. There is also evidence that for NHS111 the autocorrelations are more short-lived (I.e. mostly on day 1). For EDSS influenza-like illness the autocorrelations spread a longer time span.

Automated detection systems such as RAMMIE need to be necessarily conservative (avoiding false negatives). The figures above indicate some temporal autocorrelation in the RAMMIE-derived residuals, and hence the number of statistical alarms will be greater than if this autocorrelation did not exist. This conservative approach is logical within an operational syndromic surveillance system. This contrasts to a more traditional epidemiological study where a significant result holds much greater prominence and most effort goes into reducing false positives. In an operational system "alarms" are only the first step in a long risk assessment process [16] through which only around 1 in a 1000 will result in public health action. This is the point at which reducing false positives is emphasized. There are other practical reasons why autocorrelation is difficult to take into account. An operational syndromic surveillance system needs to predict future activity and prediction intervals weeks ahead. Incorporating an autocorrelation term into models is hence challenging as the future activity is unknown.

References

[1] Vynnycky, E., White, R.: An Introduction to Infectious Disease Modelling. Oxford University Press

[2] Brauer, F.: Modeling Influenza: Pandemics and Seasonal Epidemics. In: Brauer, F., van den Driessche, P., Wu, J. (eds.) Mathematical Epidemiology, pp. 321–347. Springer, Berlin (2008). Chap. 12

[3] Teunis, P.F.M., Chappell, C.L., Okhuysen, P.C.: *Cryptosporidium* Dose Response Studies: Variation Between Isolates. Risk Anal **22**, 175–183 (2002)

[4] Hunter, P., de Sylor, M., Risebro, H., Nichols, G., Kay, D., Hartemann, P.: Quantitative Microbial Risk Assessment of *Cryptosporidiosis* and *Giardiasis* from Very Small Private Water Supplies. Risk Anal **31**, 228–236 (2002)

[5] Fraser, C., Donnelly, C., Cauchemez, S., Hanage, W., Van Kerkhove, M., Hollingsworth, T., Griffin, J., Baggaley, R., Jenkins, H., Lyons, E., Jombart, T., Hinsley, W., Grassly, N., Balloux, F., Ghani, A., Ferguson, N., Rambaut, A., Pybus, O., Lopez-Gatell, H., Alpuche-Aranda, C., Chapela, I., Zavala, E., Guevara, D., Checchi, F., Garcia, E., Hugonnet, S., Roth, C., Collaboration, W.R.P.A.: Pandemic potential of a strain of influenza A (H1N1): early findings. Science **324**, 1557–1561 (2009)

[6] Lessler, J., Reich, N., Cummings, D., of Health, N.Y.C.D., Team, M.H.S.I.I., HP, H.N., Jordan, H., Thompson, N.: Outbreak of 2009 pandemic influenza A (H1N1) at a New York City school. N Engl J Med **361**, 2628–2636 (2009)

[7] White, L., Wallinga, J., Finelli, L., Reed, C., Riley, S., Lipsitch, M., Pagano, M.: Estimation of the reproductive number and the serial interval in early phase of the 2009 influenza A/H1N1 pandemic in the USA. Influenza Other Respir Viruses **3**, 267–276 (2009)

[8] Yang, Y., Sugimoto, J., Halloran, M., NE, N.B., Chao, D., Matrajt, L., Potter, G., E, E.K., Longini, I.: The transmissibility and control of pandemic influenza A (H1N1) virus. Science **326**, 729–733 (2009)

[9] Puleston, R., Mallaghan, C., Modha, D., Hunter, P., Nguyen-Van-Tam, J., Regan, C., Nichols, G., Chalmers, R.: The first recorded outbreak of cryptosporidiosis due to *Cryptosporidium cuniculus* (formerly rabbit genotype), following a water quality incident. J Water Health **12**, 41–50 (2014)

[10] Hagen, R.M., Loderstaedt, U., Frickmann, H.: An evaluation of the potential use of *Cryptosporidium* species as agents for deliberate release. J R Army Med Corps **160**, 289–94 (2014)

[11] Dorevitch, S., DeFlorio-Barker, S., Jones, R.M., Liu, L.: Water quality as a predictor of gastrointestinal illness following incidental contact water recreation. Water Res **83**, 94–103 (2015)

[12] Ridderstedt, F., Widerström, M., Lindh, J., Lilja, M.: Sick leave due to diarrhea caused by contamination of drinking water supply with *Cryptosporidium hominis* in Sweden: a retrospective study. J Water Health **In Press**, 1–7 (2017)

[13] Willocks, L., Crampin, A., Milne, L., Seng, C., Susman, M., Gair, R., Mousdale, M., Shafi, S., Wall, R., Wiggins, R., Lightfoot, N.: A large outbreak of cryptosporidiosis associated with a public water supply from a deep chalk borehole. Outbreak Investigation Team. Commun Dis Public Health **1**, 239–243 (1998)

[14] Pebody, R., McLean, E., Zhao, H., Cleary, P., Bracebridge, S., Foster, K., Charlett, A., Hardelid, P., Waight, P., Ellis, J., Bermingham, A., Zambon, M., Evans, B., Salmon, R., McMenamin, J., Smyth, B., Catchpole, M., Watson, J.: Pandemic Influenza A (H1N1) 2009 and Mortality in the United Kindgom: Risk Factors for Death, April 2009 to March 2010. Eurosuveillance **15**, 1–11 (2010)

[15] Morbey, R.A., Elliot, A.J., Charlett, A., Verlander, N.Q., Andrews, N., Smith, G.E.: The application of a novel "rising activity, multi-level mixed effects, indicator emphasis" (RAMMIE) method for syndromic surveillance in England . Bioinformatics **31**, 3660–3665 (2015)

[16] Smith, G.E., Elliot, A.J., Ibbotson, S., Morbey, R.A., Edeghere, O., Hawker, J., Catchpole, M., Endericks, T., Fisher, P., McCloskey, B.: Novel public health risk assessment process developed to support syndromic surveillance for the 2012 Olympic and Paralympic Games. J Public Health (Oxf), 1–7 (2016)
